# Neuronal gating of tactile input and sleep in 10-month-old infants at typical and elevated likelihood for autism spectrum disorder

**DOI:** 10.1038/s41598-022-18018-w

**Published:** 2022-08-19

**Authors:** Anna De Laet, Elena Serena Piccardi, Jannath Begum-Ali, Tony Charman, Mark H. Johnson, Emily J. H. Jones, Rachael Bedford, Teodora Gliga, The STAARS Team, Mary Agyapong, Tessel Bazelmans, Leila Dafner, Mutluhan Ersoy, Amy Goodwin, Rianne Haartsen, Hanna Halkola, Alexandra Hendry, Rebecca Holman, Sarah Kalwarowsky, Anna Kolesnik-Taylor, Sarah Lloyd-Fox, Luke Mason, Nisha Narvekar, Greg Pasco, Laura Pirazzoli, Chloë Taylor

**Affiliations:** 1 School of Psychology, University of East Anglia, Norwich Research Park, Norwich, NR4 7TJ UK; 2 Centre for Brain and Cognitive Development, Department of Psychological Sciences, Birkbeck, University of London, London, UK; 3 School of Psychology, Department of Psychological Sciences, University of East London, London, UK; 4 Psychology Department, Institute of Psychiatry, Psychology and Neuroscience, King’s College London, London, UK; 5 Department of Psychology, University of Cambridge, Cambridge, UK; 6 Department of Psychology, University of Bath, Bath, UK; 7 Department of Psychology, Kastamonu University, Kastamonu, Turkey; 8 Department of Experimental Psychology, University of Oxford, Oxford, UK; 9 Boston Children’s Hospital Harvard Medical School, Boston, USA

**Keywords:** Psychology, Circadian rhythms and sleep, Sensory processing

## Abstract

Sleep problems in Autism Spectrum Disorder (ASD) emerge early in development, yet the origin remains unclear. Here, we characterise developmental trajectories in sleep onset latency (SOL) and night awakenings in infants at elevated likelihood (EL) for ASD (who have an older sibling with ASD) and infants at typical likelihood (TL) for ASD. Further, we test whether the ability to gate tactile input, using an EEG tactile suppression index (TSI), associates with variation in SOL and night awakenings. Parent-reported night awakenings and SOL from 124 infants (97 at EL for ASD) at 5, 10 and 14 months were analyzed using generalized estimating equations. Compared to TL infants, infants at EL had significantly more awakenings and longer SOL at 10 and 14 months. The TSI predicted SOL concurrently at 10 months, independent of ASD likelihood status, but not longitudinally at 14 months. The TSI did not predict night awakenings concurrently or longitudinally. These results imply that infants at EL for ASD wake up more frequently during the night and take longer to fall asleep from 10 months of age. At 10 months, sensory gating predicts SOL, but not night awakenings, suggesting sensory gating differentially affects neural mechanisms of sleep initiation and maintenance.

## Introduction

Disturbed sleep can have profound effects on everyday life. In individuals with Autism Spectrum Disorder (ASD), sleep difficulties are highly prevalent^1,2^ and have an early onset in development. Longitudinal studies of infants at elevated familial likelihood of developing ASD (henceforth EL) suggest that sleep difficulties are present during the first year of life, before the emergence of clinical symptoms^3,4^. Understanding the origins and mechanisms that underlie sleep problems is important because poor sleep exacerbates behavioural manifestations of ASD, such as increasing communication difficulties or stereotypic behavior^5,6^, and decreasing cognitive performance^7^. In addition, there is evidence that caregiver’s well-being is impacted by poor sleep, which may in turn amplify infant sleep difficulties, resulting in decreased sleep duration and increased night awakenings^8,9^. Given the wide-ranging developmental correlates of poor sleep in infancy, such as reduced executive functioning^10^, smaller hippocampal volumes^3^, reduced social and cognitive skills^4^, and increased mental health difficulties^11^, there is a strong need to understand the developmental origins of sleep problems in neurodevelopmental disorders.

Falling asleep requires more than just closing your eyes. It needs a coordinated suppression of activity of wake-promoting neural populations across the brain. The transition to sleep depends upon the interaction between GABA-ergic sleep-promoting neurons, silencing key arousal systems located in the brainstem, posterior and lateral hypothalamus and basal forebrain, the accumulation of endogenous molecules during wakefulness that regulate homeostatic sleep drive, such as cytokines and adenosine, and circadian rhythmicity^12^. These neurobiological mechanisms are in turn influenced by environmental, behavioural and psychological factors, like light exposure^13^, exercise^14^ and stress^15^.

Studying infants at EL for developing ASD (e.g., those that have an older sibling with a diagnosis of ASD^16^) enables the prospective investigation of sleep difficulties *as they emerge*. Of those infants, around 20% will be diagnosed with ASD themselves and another 30% will exhibited subclinical symptoms of ASD at 3 years^16,17^. Studying infants at familial risk for ASD provides the opportunity to investigate atypicality manifested in the broad autism phenotype. Sleep problems in infants at EL have been described within the first year of life. In typical development, the first year of life is characterized by notable changes in sleep patterns, with infants gradually taking less time to fall asleep and waking up less at night^18,19^. In contrast, MacDuffie et al.^3^ found that parents of 6- to 12-month-old infants at EL who went on to develop ASD reported sleep onset problems. Nguyen et al.^20^ found that more night awakenings predicted ASD symptoms in infants at EL, although there was no association with sleep onset time. Begum-Ali et al.^4^ showed that a composite measure of night sleep (capturing sleep onset problems, night sleep duration and frequency of night wakings) indicated worse sleep at 5-, 10- and 14 months-of-age for infants at EL and in particular for the subgroup of infants that were diagnosed with ASD at three years. Thus, while sleep atypicalities seem to be present in the first year of life in infants at EL for ASD, the biological origins remains unclear.

Multiple possible biological causes for sleep problems in ASD have been identified, most likely overlapping and with amplifying effects^21,22^ : (1) Aberrant synaptic functionality—Sleep is highly dependent on normal synaptic functioning and synaptogenesis and synaptic plasticity rely in turn on good sleep^23,24^, (2) abnormal sleep-regulating hormones, such as atypical melatonin production^25^, (3) circadian rhythmicity disruptions, such as mutations in in core clock genes^21^, as well as (4) sensory dysregulation. One plausible mechanism, related to sensory dysregulation, which could result in sleep difficulties is atypical gating of sensory input. A network of sub-cortical structures typically ensures that the processing of sensory stimuli is attenuated, allowing sleep to be intitiated and maintained^26^. Sensory sensitivities are a core diagnostic feature of ASD^27^ and sensory processing atypicalities are common in the early development in infants at EL for ASD^29^ and infants who later develop ASD^30^. For example, when pairs of stimuli are presented with fixed inter-stimulus intervals, the response to the second stimulus, which is highly predictable and therefore less relevant for the individual, is typically attenuated—a phenomenon known as repetition suppression^31^. Infants at EL aged 8 to 10 months show reduced neural repetition suppression to repeated stimulation of auditory^32,33^—and tactile stimuli^34^. Infants at typical likelihood (TL) for ASD, but not infants at EL, demonstrate a decrease in oscillatory alpha-band activity desynchronization in response to a second stimulus with respect to a (identical) first, indexing a reduction in neural responses to repeated stimulation^34^. Attenuated repetition suppression is believed to reflect aberrant sensory gating mechanisms and is therefore particularly relevant to the study of difficulties in initiating and maintaining sleep. However, no study has yet investigated a link between these factors in early development. Correlational studies have described associations between behavioral manifestations of hypersensitivity, one potential consequence of reduced gating of sensory input, and sleep difficulties. In children and adolescents with ASD, parental reports of sensory hypersensitivity associate with delayed sleep onset latency and more night awakenings^35,36^.

As previous literature has documented specific links between sensitivity in the tactile domain and sleep disturbances, both in autistic children^36^ and in typically developing toddlers^37^, in the current study we examine whether decreased neural repetition suppression to tactile stimulation, measured at 10 months of age, associates with parent-report measures of sleep in infants at TL and EL for ASD. Sensory profiles based on caregiver reports are limited in their accuracy and do not always correlate with clinical observations^38^. In the current study, therefore, we use electroencephalography (EEG) to provide an objective measure of sensory processing atypicality.

Our first aim is to characterize the onset of sleep atypicality in infants at EL. We focus, in particular, on developmental changes in infants’ ability to initiate and maintain sleep by looking at *sleep onset latencies* and *number of night awakenings* at 5, 10, and 14 months of age and testing the point at which EL and TL sleep trajectories diverge. Differences between infants at TL and EL for ASD could result both from genetic differences between the two groups as well as from environmental differences, e.g. having an older sibling with ASD may disturb their sleep patterns. The second key aim is to test, for the first time, whether an EEG marker of tactile repetition suppression is associated with individual variation in sleep trajectories in infants at TL and EL for ASD. If such association exists, it would support the hypothesis that sleep atypicalities in infants with EL for ASD are intrinsically driven, rather than a result of their environment. Finally, we will test whether this association holds even when the group of infants that went on to develop ASD are removed. An affirmative answer will be in line with previous work suggesting the link between sensory issues and sleep is not specific to ASD^37^.

## Methods and materials

### Participants

One hundred and twenty four infants took part in a longitudinal study running from 2013 to 2019 at 5, 10, and 14 months. The experimental protocol was approved by the National Research Ethics Service (13/LO/0751) and the Research Ethics Committee of the Department of Psychological Sciences, Birkbeck, University of London (13/1617). All experiments were performed in accordance with relevant guidelines and regulations. Parents provided informed, written consent before the onset of the study. The study recruited participants with a first degree relative with ASD and/or ADHD. As the focus of the current study is understanding ASD-related atypicalities in sensory processing and sleep, infants with a family history of only ADHD were not included. Participants were classified as infants at EL for ASD if they had a first-degree relative diagnosed with ASD by a licensed clinician (*n* = 97, female = 45). Infants with no first-degree relatives with an ASD diagnosis and a typically developing older sibling were classified as infants at TL for ASD (*n* = 27, female = 9). Infants at TL were recruited from a volunteer database at the Centre for Brain and Cognitive Development, Birkbeck University of London (See Supplementary materials 1.1 for more information on participant recruitment and diagnosis). At the 10-month visit, infants (*n* = 65; EL = 48, TL = 17), participated in an EEG study measuring responses to tactile stimulation. The sample size for particular analyses varied due to missing responses or attrition (see Tables 1 and S1).

**Table 1:** Characteristics of participants included in data analysis at 5-, 10- and 14-month assessments.

	EL	TL	*ρ*-value
**5 month visit**			
Age in days	176 (20)	179 (14)	0.463^a^
Number of awakenings	2.00 (1.48)	2.44 (1.47)	0.209^a^
N	65	25	
M:F	35:30	18:7	0.117^b^
Sleep onset latency in min	12.42 (10.35)	11.15 (10.10)	0.604^a^
N	65	24	
M:F	34:31	17:7	0.117^b^
**10 month visit**			
Age in days	319 (15)	322 (17)	0.430^a^
Number of awakenings	1.95 (1.37)	1.27 (1.08)	**0.032** ^a^
N	81	22	
M:F	46:35	15:7	0.335^b^
Sleep onset latency in min	11.45 (7.51)	7.92 (6.19)	**0.041**^a^
N	82	22	
M:F	46:36	15:7	0.307 ^b^
Tactile Supression Index	− 0.009 (0.212)	0.141 (0.141)	**0.008**^a^
N	51	17	
M:F	24:27	10:7	0.401^b^
**14 month visit**			
Age in days	450 (19)	448 (18)	0.548^a^
Number of awakenings	1.87 (1.46)	1.00 (1.45)	**0.022** ^a^
N	83	19	
M:F	44:39	12:7	0.423^b^
Sleep onset latency in min	13.18 (11.40)	6.46 (7.25)	**0.002**^a^
N	84	19	
M:F	45:39	12:7	0.448^b^

Means (standard deviation); ^a^ independent t-test; ^b^ Pearson Chi square test.

Significant values are in [bold].

### Measures

#### Sleep measures

 Questions from the Sleep and Settle Questionnaire (SSQ; 31) were used as measures for sleep onset latency and the number of awakenings. The number of awakenings indicates the number of times the infant woke up during the night on average in the preceding week. Integers were required for analysis of this ordinal variable (see Analytical approach), therefore where parents filled in a range instead of one number, the average was taken and, in case of a non-integer, the value was truncated (e.g. 2.5 would become 2). Parents reported separately on the time it took to settle their infant for day (5am to 6 pm), evening (6 pm to 10 pm) and night sleeps (10 pm to 5am), again, with an average estimate over the preceding week. If parents reported a range, the mean was taken. We averaged day, evening and night values to create a continuous sleep onset latency measure. Participants were also included if they only filled in one or two of the questions, since not all infants take two additional naps. However, this was less than 10% of the total participants included in the calculation of sleep onset latency at all three visits.

#### ASD diagnosis

 At 3 years, infants at EL were assigned a best estimate research diagnosis ASD (EL-ASD +) or non-autism (EL-ASD-) according to the DSM-5 diagnostic criteria by experienced researchers with the help of a licensed clinical psychologist (GP and TC). The decision was based on outcomes from the Autism Diagnostic Observation Schedule, Second Edition (ADOS-2; 32), Autism Diagnostic Interview-Revised (ADI-R; 33), Mullen Scales of Early Learning (MSEL; 34) assesments, the Vineland Adaptive Behaviour Scales (VABS; 35) and researcher observations during previous visits. (SM 1.1).

#### EEG Paradigm

 A full description is reported in Piccardi et al.^34^.

##### Stimuli

**Figure 1:**
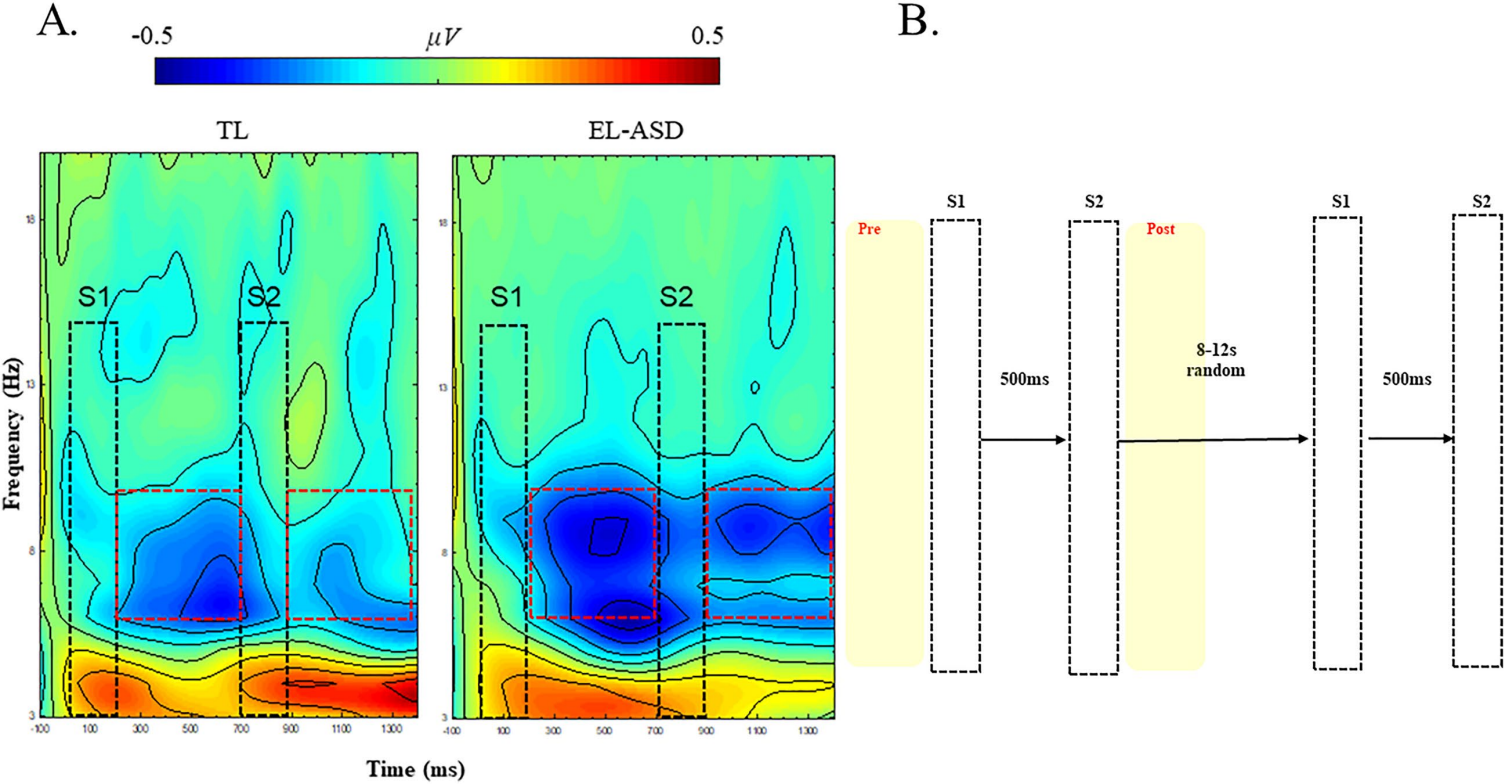
(**A**) Time frequency plots in both groups, TL and EL. Black dotted lines indicate the first (S1) and second (S2) stimuli. Red dotted lines indicate the 500-ms-long time-windows post-stimulus offset selected for statistical analysis. Amplitude scale is − 0.5, 0.5μv. (**B**) Experimental design. Vibrotactile stimuli are presented in pairs (S1 and S2) with a fixed interstimulus interval of 500 ms. The interval between the onsets of pairs of stimuli ranged from 8 to 12 s randomly. Figures adapted from Piccardi et al. (2021) and created using WTools^39^.

Custom built voice coil tactors were attached to the bare soles of each foot of the infant with cohesive tape. Vibrotactile stimuli were delivered to both feet simultaneously with a frequency of 220 Hz. Stimuli lasted 200 ms and were consistently presented in pairs (S1–S2) with a 500 ms interstimulus interval (Fig. 1). The time between pairs of stimuli, the intertrial interval, varied randomly between 8 and 12 s. In total, 38 pairs of stimuli were administered split across two 4 min blocks with a 2 min break in between. As a distraction, a visually engaging cartoon without language content (Fantasia by Walt Disney) was played during the experiment. Infants were seated on the lap of the parent 60 cm from the screen in a dimly illuminated room.

##### Apparatus and time–frequency analysis of EEG

EEG was recorded using 124 channels of a 128-channel HydroCel Geodesic Sensor Net connected to a NetAmps 400 amplifier (Electrical Geodesic, Eugene, OR) and referenced online to the vertex (Cz). Net station (Electrical Geodesic) was used to pre-process the EEG data offline. If individual epochs exhibited voltage changes over 200 µV in one segment (identified by automated artifact detection), individual channels within segments were eliminated after additional visual inspection. Artifact free EEG segments were processed and analysed using EEGLAB (v.13.4.3b) in MATLAB®. Spectral decompositions were conducted using *Wtools* (developed by E. Parise, L. Filippin, & G. Csibra, available upon request)*,* employing complex Morlet wavelets 3-20 Hz with 1 Hz resolution*.* A continuous wavelet transformation of all segments was conducted, and the absolute value of the results was extracted. A 100 ms pre-stimulus window was used as a baseline. Individual epochs were averaged per participant. Time–frequency decomposition was used to quantify oscillatory alpha amplitude desynchronization to tactile stimulation (i.e. 6–10-Hz alpha amplitude during the task as compared to alpha amplitude at baseline). The average 6–10-Hz alpha desynchronization oscillatory amplitude was extracted from two 500-ms-long windows time-locked to S1 and S2 offset. A tactile suppression index (TSI) was computed by subtracting alpha amplitude desynchronization at S2 from alpha amplitude desynchronization at S1.

### Analytical approach

Statistical analysis was performed in SPSS v25. Three values of sleep onset latency (2 EL; 1 TL), one value of the number of awakenings (TL) and one value of the TSI (EL) were more than three standard deviations above the mean and trimmed to one integer above the highest value. To analyse the trajectory of sleep onset latency and the number of awakenings, generalized estimating equations (GEE) were used factoring in Group and time of the measurement (Visit). GEE was chosen to model the non-normal response variables and to accommodate for missing data. For number of awakenings, a count variable, a Poisson distribution with a log-link was specified. Due to a right skew for the sleep onset latency variable (see Table S2 for skewness and normality), a gamma distribution with log-link was specified. An integer of 1 was added to the sleep onset latency variable, so values of zero would not be omitted in the GEE. Maximum likelihood was selected for scale parameter estimation. The structure of the working correlation matrix was specified as ‘unstructured’ with a robust estimator.

To assess the main effects of Visit (5, 10 and 14 months) and Group (EL and TL), the GEE was run with main effects only and then a Group*Visit interaction was added in a separate step, from which the interaction terms is ascertained. Post hoc Bonferroni corrected pairwise comparisons were run for significant main effects of Visit. In case of a significant interaction with Visit, a separate GEE was run per timepoint to assess group differences. GEE models were run to test whether the TSI was associated with the sleep parameters, first concurrently (i.e. at 10 months), then longitudinally. To test the generalisability of results across the EL group, all analyses were repeated after the removing infants who were subsequently diagnosed with ASD at 36 months. We also excluded infants at EL who did not come in to the 36 month assessment and therefore could not be assigned to EL-ASD + nor EL-ASD-.

## Results

### Sleep trajectory

**Figure 2:**
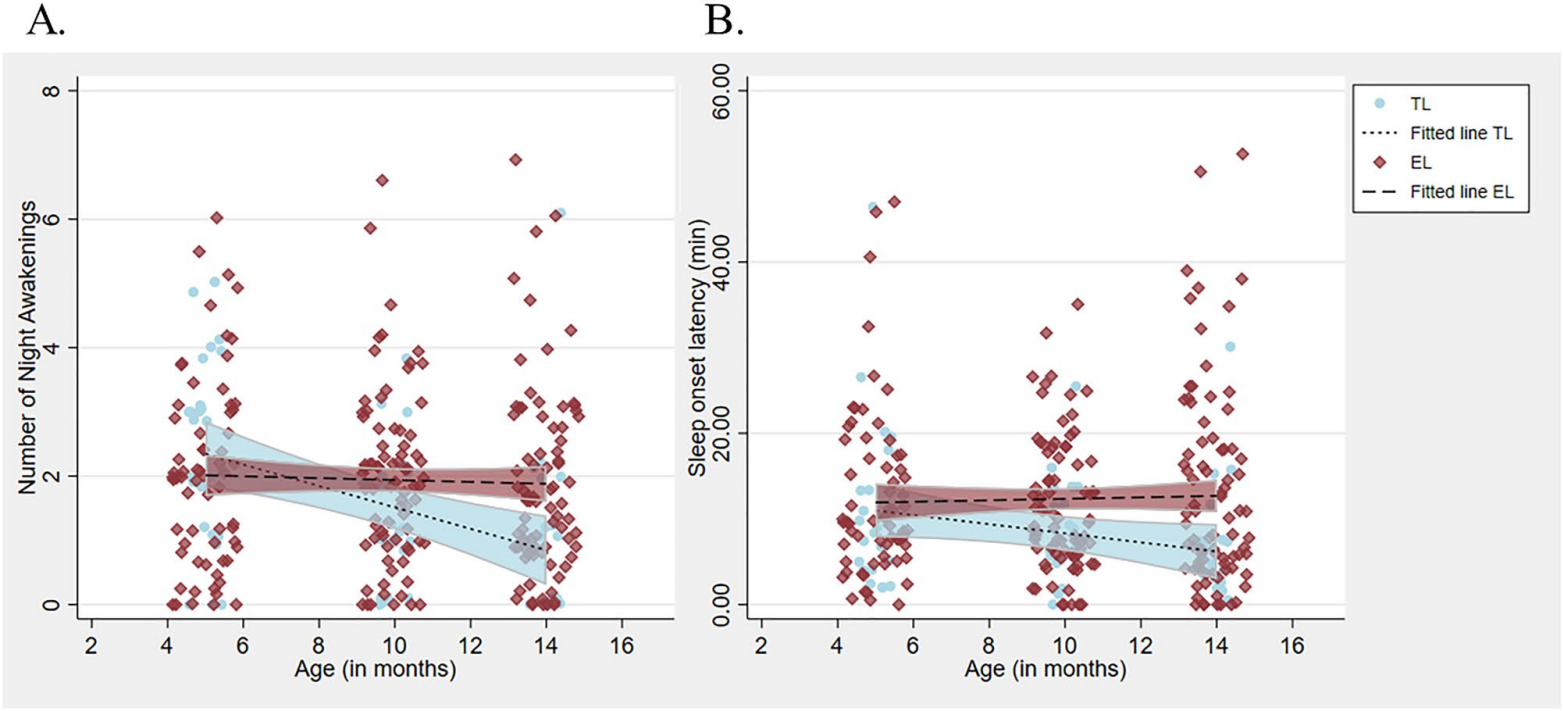
Sleep trajectories from 5 to 14 months. Infants at typical likelihood for ASD (TL) are depicted in blue and infants at elevated likelihood for ASD (EL) are depicted in red. Fitted lines are shaded by the 95% confidence interval. (**A**) The number of night awakenings. (**B**) Sleep onset latency.

The number of awakenings significantly decreased each consecutive visit in the whole sample (see Fig. 2, Waldχ^2^ = 10.503, *p* = 0.005) with post hoc pairwise comparisons indicating that infants had significantly fewer awakenings at 14 months (estimated mean [EM] = 1.63; 95% confidence interval [CI] = 1.36, 1.97) compared to 5 months (EM = 2.14; CI = 1.84, 2.50; *p* = 0.004, Bonferroni corrected *p* = 0.05/3). There was no significant main effect of ASD likelihood status on awakenings (Waldχ^2^ = 1.116, *p* = 0.291), but there was a significant interaction between visit and ASD likelihood status (Waldχ^2^ = 10.777, *p* = 0.005); post-hoc tests revealed no significant group difference in awakenings at 5 months (Waldχ^2^ = 1.773, *p* = 0.183), but a significant difference at 10 months with infants at EL waking more often (Waldχ^2^ = 5.068, *p* = 0.024), and a marginally significant difference, in the same direction, at 14 months (Waldχ^2^ = 3.465, *p* = 0.063).

For sleep onset latency, there was no significant main effect of visit (Waldχ^2^ = 2.567, *p* = 0.277), nor did ASD likelihood status reach significance (Waldχ^2^ = 2.850, *p* = 0.091). However, the interaction between visit and ASD likelihood status was significant (Waldχ^2^ = 17.421, *p* < 0.001). Post-hoc tests showed no significant differences at 5 months (Waldχ^2^ = 0.273, *p* = 0.601), but significantly longer sleep onset latencies at 10 months in infants at EL compared to infants at TL (Waldχ^2^ = 4.402, *p* = 0.036), which persisted at 14 months (Waldχ^2^ = 7.545, *p* = 0.006).

Looking at the sleep trajectory by group (see Fig. 2), the number of awakenings (Waldχ^2^ = 13.239, *p* = 0.001) and sleep onset latency (Waldχ^2^ = 15.272, *p* < 0.001) significantly decreased with age in infants at TL, while in infants at EL they remained stable over time (Waldχ^2^ = 1.753, *p* = 0.416 and Waldχ^2^ = 3.787, p = 0.151, respectively). In TL infants, post hoc tests indicated that the number of awakenings was significantly higher at 5 months (EM = 2.47; CI = 1.97, 3.09) compared to 10 months (EM = 1.36; CI = 0.94, 1.97; *p* = 0.001, Bonferroni corrected) and 14 months (EM = 1.09; CI = 0.64, 1.86; *p* < 0.001, Bonferroni corrected). TL infants’ sleep onset latency also significantly decreased from 5 months (EM = 12.62 min; CI = 9.28 min, 17.16 min) to 14 months (EM = 7.93 min; CI = 5.53 min, 11.37 min; *p* = 0.001 Bonferroni corrected), but not from 5 to 10 months (EM = 7.94 min; CI = 5.45 min, 11.59 min; *p* = 0.096). Infants at EL (*n* = 82) were assessed for ASD at a subsequent 36-month visit; 12 infants met diagnostic criteria for ASD (EL-ASD +) and 70 infants did not (EL-ASD-) (see Table S3 for descriptives).

When the sleep trajectory analyses were re-run by splitting the data in groups based on ASD outcome (TL vs EL-ASD- and TL vs EL-ASD +), the small EL-ASD + group (*n* = 12) tended to have the most extreme values. In terms of sleep onset latencies, infants at EL-ASD + took significantly longer to fall asleep than infants at TL at 10 (Waldχ^2^ = 8.185, *p* = 0.004) and 14 months (Waldχ^2^ = 11.093, *p* = 0.001), while infants at EL-ASD- had significantly longer sleep onset latencies than infants at TL at 14 months only (Waldχ^2^ = 6.064, *p* = 0.014), but not at 10 months (Waldχ^2^ = 1.791, *p* = 0.181). For night awakenings, infants at EL-ASD + had significantly more night awakenings than infants at TL at 14 months (Waldχ^2^ = 7.252, *p* = 0.007), but not at 10 months (Waldχ^2^ = 2.021, *p* = 0.155), while this was the opposite for infants at EL-ASD- (At 10 months: Waldχ^2^ = 4.663, *p* = 0.031; At 14 months: Waldχ^2^ = 2.563, *p* = 0.109) (Table S4-5 and Figures S1-2).

### Associations with tactile repetition suppression

**Figure 3:**
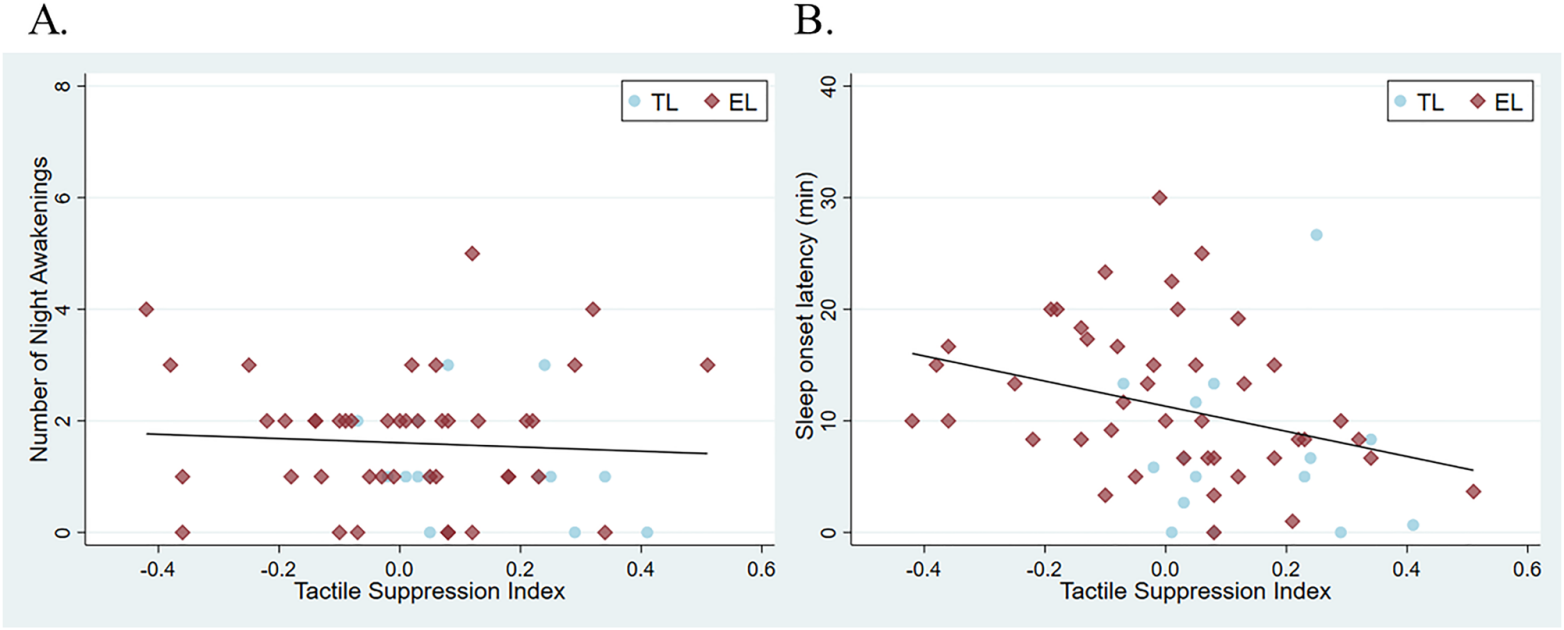
Associations with tactile suppression index (TSI) at the 10-month visit. Infants at typical likelihood for ASD (TL) are depicted in blue and infants at elevated likelihood for ASD (EL) are depicted in red. (**A**) The number of night awakenings is not significantly associated with TSI. (**B**) Sleep onset latency is significantly associated with TSI.

Scores on the TSI were significantly higher (decreased attenuation of response with repetition) in infants at TL compared to infants at EL (t = 2.717 (66), *p* = 0.008) in line with results previously reported by Piccardi et al.^25^ in infants from the same cohort (Table 1). Summary correlations between TSI and sleep measures at 10 and 14 months are presented in Table 2. To test whether emerging sleep problems associate with the TSI concurrently, two GEE models were run, one with the number of awakenings and the other with sleep onset latency at 10 months as the outcome variable. In both models, TSI and ASD likelihood status were entered as predictors. TSI had a significant effect on sleep onset latency (Waldχ^2^ = 7.775, *p* = 0.005), but not on the number of awakenings (Waldχ^2^ = 0.009, *p* = 0.923), see Fig. 3. ASD likelihood status did not have a significant effect on awakenings at 10 months (Waldχ^2^ = 2.616, *p* = 0.106) and there was also no significant effect of the the interaction between ASD likelihood status and TSI (Waldχ^2^ = 1.146, *p* = 0.284). ASD likelihood status did not have a significant effect (Waldχ^2^ = 2.125, *p* = 0.145) nor did it significantly interact with TSI (Waldχ^2^ = 0.284, *p* = 0.594) in predicting sleep onset latency at 10 months.

**Table 2:** Correlation coefficients (Spearman) of all measures used in the analyses.

	TSI	Awakenings 5mo	Awakenings 10mo	Awakenings 14mo	SOL 5mo	SOL 10mo	SOL 14mo
TSI	1.000						
Awakenings 5mo	0.107	1.000					
Awakenings 10mo	− 0.075	0.379**	1.000				
Awakenings 14mo	− 0.030	0.386**	0.577**	1.000			
SOL 5mo	− 0.348*	0.208*	0.209	0.172	1.000		
SOL 10mo	− 0.370**	0.042	0.354**	0.360**	0.426**	1.000	
SOL 14mo	− 0.260*	0.190	0.288**	0.429**	0.469**	0.631**	1.000

*Significant correlation *p* < .05 (2-tailed). **Significant correlation *p* < .01 (2-tailed).

*SOL* Sleep Onset Latency, *mo* months.

Significant values are in [bold].

To evaluate if TSI at 10 months associates longitudinally with the sleep parameters at 14 months, over and above 10-month sleep, separate models were run with 14-month sleep onset latency or number of awakenings as outcomes. In both models TSI, ASD likelihood status and the relevant 10-month sleep measure (onset latency/number of awakenings), were entered as predictors. TSI did not significantly associate with the number of awakenings (Waldχ^2^ = 0.128, *p* = 0.721) or sleep onset latency at 14 months (Waldχ^2^ = 0.635, *p* = 0.425). The results remained substantively similar when the models were re-run excluding the EL-ASD + participants (*n* = 5), suggesting that these infants did not drive the results in the main analysis and that the association between TSI and sleep is not specific to ASD (see Table S6 and S7).

## Discussion

Characterising sleep trajectories of infants at TL and EL for ASD revealed that sleep onset latency and night awakenings decrease in infants at TL from 5 to 14 months. These patterns mirror previous findings that sleep consolidates during the first year of life in typically developing infants^40^. In contrast, no developmental change was seen in infants at EL, leading to significant differences between the groups from 10 months, with longer sleep onset latency and more night awakenings in infants at EL than TL. Further, our results show that an objective measure of poor sensory gating of tactile stimulation significantly associates with longer sleep onset latency. This finding was independent of ASD likelihood status, implying that there is a general association between sensory gating and sleep onset latency, in line with previous evidence in typically developing children^37^. No association between sensory gating and number of night awakenings was found, either suggesting a differential mechanism of sensory gating during pre-sleep wake and sleep itself or simply reflecting unreliable caregiver reports of night awakenings compared to sleep onset latency. We discuss each of these findings in turn, below.

### Trajectories of sleep parameters

In a previous paper, using the same cohort of children, we reported that a composite score of night sleep was worse in infants at EL compared to infants at TL at 5, 10 and 14 months^4^. Here, we specifically focused on two measures of sleep expected to be influenced by the ability to gate sensory input—sleep onset latency and night awakenings. Our findings suggest differences in both of these parameters emerge between 5 and 10 months, which narrows down the developmental interval within which to investigate underlying causes. Using a less precise measure, asking about the presence of frequent night awakenings (3 or more) and not the exact number awakenings, in a longitudinal population study, Humphreys et al.^41^ only found a significant difference in frequent night awakenings between TD and ASD at 30 months, but not at 6 or 18 months. In our sample, however, infants at EL woke up more frequently than infants at TL from 10 months, suggesting an earlier emergence.

Subdividing the EL infants into infants that were or were not subsequently diagnosed with ASD (EL-ASD + and EL-ASD- respectively) showed that infants at EL-ASD + took longest to fall asleep, suggesting that sleep onset latency is intrinsically driven in infants at EL for ASD, rather than a result of a shared environment with an older sibling with ASD. The differences in night awakening trajectories between infants at EL-ASD + and EL-ASD- were less consistent, likely due to the small sample sizes in the subgroups. Further research is needed to investigate these differences in infants at EL-ASD + and EL-ASD-.

### The impact of sensory gating on sleep

The fact that reduced sensory gating was associated with longer sleep onset latency, but not with more night awakenings is consistent with literature suggesting these sleep processes have different underlying mechanisms. For example, adults with sleep onset problems, showed reduced repetition suppression to auditory stimuli during pre-sleep wake compared to good sleepers, but not during rapid eye movement (REM) or non REM2 (N2)^42^. In support of different neural mechanisms underlying sleep onset and maintenance, the manipulation of the inhibitory neurotransmitter GABA_A_ receptor, which has an important role in sleep^43^, affected sleep initiation more than sleep maintenance in fruit flies^44^. In adult patients with primary insomnia (PI), auditory stimulation did not increase the number of awakenings compared to an undisturbed, baseline night sleep. However, the PI group was more likely to stay in REM sleep when stimulation occurred while controls transitioned to N2 more often^45^. Thus, while increased stimulus input (due to poor gating) might not result in more awakenings, it might still affect sleep quality and architecture to a larger extent in populations with sleep difficulties, like in ASD. The incongruity of the reported association between sensory gating and sleep onset latency but not awakenings might also reflect the nature of the sensory gating measure used in this study. Sensory gating was measured during wakefulness and might therefore be more closely related to arousibility at sleep onset compared to arousibility from sleep. Kisley et al.^46^ reported differences in sensory gating, although in response to auditory stimulation, dependent on vigilance state in the same individuals. Alternatively, the discrepancy in our findings between awakenings and sleep onset latency could be caused by the accuracy of caregiver report. Sadeh et al.^47^ found that parents reported the number of awakenings significantly less accurately than sleep onset in infants when compared with actigraphy results. Moreover, Pisch et al.^48^ found no significant association between parent reported awakenings and actigraphy in infants.

The association we find between tactile repetition suppression and sleep onset latency is indicative of common underlying mechanisms. One possibility is that impaired GABAergic functioning impacts both sensory gating and sleep*.* GABA is the main inhibitory neurotransmitter in the brain. Altered functioning of GABAergic signaling in ASD is evident from lower GABA levels, reduced expression of GABAergic genes and microdeletion in genes coding for subunits of the GABA_A_ receptor^49^. Both sensory processing atypicalities and sleep onset problems could be triggered by reduced GABA levels. In fact, sleep onset latency was decreased in rats after oral administration of GABA^50^, and mutation of GABA_A_-receptor in fruit flies resulted in a reduction of sleep onset latency^44^. At the same time, Puts et al.^51^ found that reduced sensorimotor GABA-levels in children with ASD are associated with sensitivity to touch. Thus, an impaired GABA-ergic system could underlie the co-occurrence of sensory issues and sleep disturbances in ASD. In general, there is accumulating evidence to believe that an exitation/inhibition (E/I) imbalance, particularly relevant during brain development, plays an important role in the pathophysiology of ASD. Besides affecting basic sensory processing and sleep, an E/I imbalance disturbs optimal information transmission, which could alter processing of complex information such as social stimuli, resulting in social and cognitive impairments seen in ASD.

Another possibility is that atypical thalamocortical connectivity underlies both sensory and sleep problems in ASD. A recent paper found that increased sleep latency was associated with increased thalamocortical connectivity, as well as elevated BOLD activity in the cortex in children with ASD^52^.

### Longitudinal effects of sensory gating

TL participants showed a further decrease in sleep onset latency between 10 and 14 months. These changes may, in part, reflect the development of self-soothing strategies. The mechanisms driving individual progress in self-soothing are poorly understood, but it is believed this requires infants to identify body cues for sleepiness and to use behavioral strategies, such as sucking on fingers, to fall asleep more easily^53,54^. It is therefore plausible that poor sensory gating may not only delay sleep onset but it may also interfere with the development of these strategies. We found tactile repetition suppression did not predict sleep onset latency at 14 months after controlling for 10-month sleep onset latency. This suggests that the effects of reduced sensory gating on sleep do not accumulate over time. Manelis-Baram et al.^55^ found that changes in sleep disturbances between 3 and 5 years of age were associated with changes in sensory sensitivities specifically, and not with other core ASD symptoms, in autistic children. Similar to our results, they found that initial sensory profile scores did not predict sleep disturbances at a later timepoint. The absence of long lasting impacts on sleep suggest that addressing sensory issues for interventions could therefore be an effective strategy at any age.

ASD is a complex, multifaceted disorder, with sleep problems, which are equally diverse in ASD as the disorder itself, and that likely originate from multiple pathways. Our findings support one of those pathways, however other underlying mechanisms most likely contribute to sleep and sleep onset problems in ASD. Differences in melatonin production, clock gene expression and behavioural complications such as the high prevalence of anxiety that is associated with ASD, have all been suggested as contributory factors to sleep problems^22^.

While this study is novel in its use of an objective measure for sensory gating, sleep is captured by caregiver reports and not by more objective measures. The use of actigraphy or polysomnography could greatly improve the reliability of sleep behaviours, particularly for night awakenings.

Given the critical role of sleep to development, our finding that diminished tactile repetition suppression is associated with prolonged sleep onset has important clinical implications. While atypical sensory gating in infants at EL for ASD is not specific to the tactile domain^33^, tactile input may be particularly prominent before and during sleep compared to sensory input from other modalities. Visual input can be reduced by turning off the lights and auditory input by closing a door; in contrast, infants will experience continuous tactile input, especially when moving around in bed. This suggests that interventions which target tactile input to improve sleep may be particularly fruitful. An encouraging first step was made by a study showing that sleep quality improved in a group of children with sensory processing disorder that received a massage before bedtime^56^. Since both sensory atypicalities and sleep disturbances are common in the early development of ASD, early interventions focussed on reducing sensory input or sensitivity have the potential to alleviate sleep difficulties with positive downstream effects on cognitive development and wellbeing.

## Supplementary Information

Supplementary Information.

## Data Availability

At present, the datasets generated and/or analysed during the current study are not publicly available due to confidentiality constraints within our ethical approvals. In the future, we hope to make these datasets available via The BASIS Network (http://www.basisnetwork.org/) upon completion of the requisite data access and sharing protocols.

## References

[1] Richdale AL, Schreck KA (2009). Sleep problems in autism spectrum disorders: Prevalence, nature and possible biopsychosocial aetiologies. Sleep Med. Rev..

[2] Robinson-Shelton A, Malow B (2015). Sleep disturbances in neurodevelopmental disorders. Curr. Psychiatry Rep..

[3] MacDuffie KE (2020). Sleep onset problems and subcortical development in infants later diagnosed with autism spectrum disorder. Am. J. Psychiatry.

[4] Begum-Ali, J. *et al.* Infant sleep predicts trajectories of social attention and later autism traits. *Manuscr. Submitt. Publ.* (2022). 10.1111/jcpp.13791 PMC10952761 36991307

[5] Cohen S, Conduit R, Lockley SW, Rajaratnam SM, Cornish KM (2014). The relationship between sleep and behavior in autism spectrum disorder (ASD): a review. J. Neurodev. Disord..

[6] Mazurek MO, Dovgan K, Neumeyer AM, Malow BA (2019). Course and predictors of sleep and co-occurring problems in children with autism spectrum disorder. J. Autism Dev. Disord..

[7] Limoges É, Bolduc C, Berthiaume C, Mottron L, Godbout R (2013). Relationship between poor sleep and daytime cognitive performance in young adults with autism. Res. Dev. Disab..

[8] Sidor A, Fischer C, Eickhorst A, Cierpka M (2013). Influence of early regulatory problems in infants on their development at 12 months: a longitudinal study in a high-risk sample. Child Adolesc. Psychiatry Ment. Health.

[9] Sinai D, Tikotzky L (2012). Infant sleep, parental sleep and parenting stress in families of mothers on maternity leave and in families of working mothers. Infant Behav. Dev..

[10] Tesfaye R (2021). Investigating longitudinal associations between parent reported sleep in early childhood and teacher reported executive functioning in school-aged children with autism. Sleep.

[11] Cook F (2020). Infant sleep and child mental health: a longitudinal investigation. Arch. Dis. Child..

[12] Lim MM, Szymusiak R (2015). Neurobiology of arousal and sleep: Updates and insights into neurological disorders. Curr. Sleep Med. Rep..

[13] Tähkämö L, Partonen T, Pesonen A-K (2019). Systematic review of light exposure impact on human circadian rhythm. Chronobiol. Int..

[14] Dworak M, Diel P, Voss S, Hollmann W, Strüder HK (2007). Intense exercise increases adenosine concentrations in rat brain: Implications for a homeostatic sleep drive. Neuroscience.

[15] Lo Martire V, Caruso D, Palagini L, Zoccoli G, Bastianini S (2020). Stress and sleep: A relationship lasting a lifetime. Prenat. Stress Brain Disord. Later Life.

[16] Ozonoff S (2011). Recurrence risk for autism spectrum disorders: a Baby Siblings Research Consortium study. Pediatrics.

[17] Messinger D (2013). Beyond autism: A baby siblings research consortium study of high-risk children at three years of age. J. Am. Acad. Child Adolesc. Psychiatry.

[18] Henderson JMT, France KG, Owens JL, Blampied NM (2010). Sleeping through the night: The consolidation of self-regulated sleep across the first year of life. Pediatrics.

[19] Joseph D (2015). Getting rhythm: How do babies do it? *Arch*. Dis. Child. Fetal Neonatal Ed..

[20] Nguyen A, Murphy L, Kocak M, Tylavsky F, Pagani L (2018). Prospective associations between infant sleep at 12 months and autism spectrum disorder screening scores at 24 months in a community-based birth cohort. J. Clin. Psychiatry.

[21] Lorsung E, Karthikeyan R, Cao R (2021). biological timing and neurodevelopmental disorders: A role for circadian dysfunction in autism spectrum disorders. Front. Neurosci..

[22] Deliens G, Peigneux P (2019). Sleep–behaviour relationship in children with autism spectrum disorder: Methodological pitfalls and insights from cognition and sensory processing. Dev. Med. Child Neurol..

[23] Tononi G, Cirelli C (2014). Sleep and the price of plasticity: From synaptic and cellular homeostasis to memory consolidation and integration. Neuron.

[24] Wang G, Grone B, Colas D, Appelbaum L, Mourrain P (2011). Synaptic plasticity in sleep: Learning, homeostasis and disease. Trends Neurosci..

[25] Wu Z (2020). Autism spectrum disorder (ASD): Disturbance of the melatonin system and its implications. Biomed. Pharmacother..

[26] Gent TC, Bassetti CL, Adamantidis AR (2018). Sleep-wake control and the thalamus. Syst. Neurosci..

[27] American Psychiatric Association. *Diagnostic and statistical manual of mental disorders*. (American Psychiatric Publishing, 2013).

[28] Gliga T, Jones EJH, Bedford R, Charman T, Johnson MH (2014). From early markers to neuro-developmental mechanisms of autism. Dev. Rev. DR.

[29] Van Etten HM (2017). Increased prevalence of unusual sensory behaviors in infants at risk for, and teens with, autism spectrum disorder. J. Autism Dev. Disord..

[30] Mulligan S, White BP (2012). Sensory and motor behaviors of infant siblings of children with and without autism. Am. J. Occup. Ther..

[31] Nordt M, Hoehl S, Weigelt S (2016). The use of repetition suppression paradigms in developmental cognitive neuroscience. Spec. Issue Repetit. Suppr. Integr. View.

[32] Guiraud JA (2011). Differential habituation to repeated sounds in infants at high risk for autism. NeuroRep..

[33] Kolesnik A (2019). Increased cortical reactivity to repeated tones at 8 months in infants with later ASD. Transl. Psychiatry.

[34] Piccardi ES (2021). Behavioural and neural markers of tactile sensory processing in infants at elevated likelihood of autism spectrum disorder and/or attention deficit hyperactivity disorder. J. Neurodev. Disord..

[35] Mazurek MO, Petroski GF (2015). Sleep problems in children with autism spectrum disorder: examining the contributions of sensory over-responsivity and anxiety. Sleep Med..

[36] Tzischinsky O (2018). Sleep disturbances are associated with specific sensory sensitivities in children with autism. Mol. Autism.

[37] Appleyard, K. *et al.* Sleep and Sensory Processing in Infants and Toddlers: A Cross-Sectional and Longitudinal Study. *Am. J. Occup. Ther.* **74**, 7406205010p1–7406205010p12 (2020). 10.5014/ajot.2020.038182 33275561

[38] Mikkelsen, M., Wodka, E. L., Mostofsky, S. H. & Puts, N. A. J. Autism spectrum disorder in the scope of tactile processing. *Autism Spectr. Cond. – Underst. Sens. Soc. Featur.* **29**, 140–150 (2018). 10.1016/j.dcn.2016.12.005 PMC5481487 28089657

[39] Parise E, Csibra G (2013). Neural responses to multimodal ostensive signals in 5-month-old infants. PLoS ONE.

[40] Henderson JMT, Blampied NM, France KG (2020). Longitudinal study of infant sleep development: Early predictors of sleep regulation across the first year. Nat. Sci. Sleep.

[41] Matthey S (2001). The sleep and settle questionnaire for parents of infants: Psychometric properties. J. Paediatr. Child Health.

[42] Lord C (2000). The autism diagnostic observation schedule—generic: A standard measure of social and communication deficits associated with the spectrum of autism. J. Autism Dev. Disord..

[43] Lord C, Rutter M, Le Couteur A (1994). Autism Diagnostic Interview-Revised: A revised version of a diagnostic interview for caregivers of individuals with possible pervasive developmental disorders. J. Autism Dev. Disord..

[44] Mullen, E. M. *Mullen scales of early learning (AGS ed)*. (American Guidance Service Inc, 1995).

[45] Sparrow, S. S. & Cicchetti, D. V. The Vineland Adaptive Behavior Scales. in *Major Psychological Assessment Instruments, Vol. 2.* 199–231 (Allyn & Bacon, 1989).

[46] Kisley MA, Olincy A, Freedman R (2001). The effect of state on sensory gating: comparison of waking REM and non-REM sleep. Clin. Neurophysiol..

[47] Sadeh A (1996). Evaluating night wakings in sleep-disturbed infants: A methodological study of parental reports and actigraphy. Sleep.

[48] Pisch, M. A longitudinal study of infant sleep and its effects on cognitive development. in (2015).

[49] Coghlan S (2012). GABA system dysfunction in autism and related disorders: from synapse to symptoms. Neurosci. Biobehav. Rev..

[50] Kim S, Jo K, Hong K-B, Han SH, Suh HJ (2019). GABA and l-theanine mixture decreases sleep latency and improves NREM sleep. Pharm. Biol..

[51] Puts NAJ (2017). Reduced GABA and altered somatosensory function in children with autism spectrum disorder. Autism Res..

[52] Linke AC (2021). Sleep problems in preschoolers with autism spectrum disorder are associated with sensory sensitivities and thalamocortical overconnectivity. Biol. Psychiatry Cogn. Neurosci. Neuroimaging.

[53] Camerota M, Propper CB, Teti DM (2019). Intrinsic and extrinsic factors predicting infant sleep: Moving beyond main effects. Dev. Rev..

[54] Goodlin-Jones BL, Burnham MM, Gaylor EE, Anders TF (2001). Night waking, sleep-wake organization, and self-soothing in the first year of life. J. Dev. Behav. Pediatr..

[55] Manelis-Baram L (2021). Sleep disturbances and sensory sensitivities co-vary in a longitudinal manner in pre-school children with autism spectrum disorders. J. Autism Dev. Disord..

[56] Spira G (2021). A sensory intervention to improve sleep behaviors and sensory processing behaviors of children with sensory processing disorders. Ir. J. Occup. Ther..

