# Supplementary material for: Neuronal gating of tactile input and sleep in 10-month-old infants at typical and elevated likelihood for autism spectrum disorder
Source: Sci Rep. 2022 Aug 19;12:14188. doi: 10.1038/s41598-022-18018-w (PMC9391390; doi:10.1038/s41598-022-18018-w)
Supplement: Supplementary file 1 — Supplementary Information. [file 41598_2022_18018_MOESM1_ESM.docx]

Supplementary materials

NEURONAL GATING OF TACTILE INPUT AND SLEEP IN 10-MONTH-OLD INFANTS AT TYPICAL AND ELEVATED LIKELIHOOD FOR AUTISM SPECTRUM DISORDER

Anna De Laet^1^, Elena Serena Piccardi^2,3^, Jannath Begum-Ali^2^, Tony Charman^4^, Mark H. Johnson^2,5^, Emily J.H. Jones^2^, Rachael Bedford^6^, Teodora Gliga^1,2^ and the STAARS Team^φ^

## 1. Methods

### 1.1 Further information on participant recruitment and clinical assessment

Participants were recruited for a longitudinal study running from 2013 to 2019 from a volunteer database, community flyers, internet adverts and clinical networks. Participant families were reimbursed expenses for travel, subsistence and overnight stay if required. Infants were given a certificate and t-shirt after each visit.

Information about diagnostic status was ascertained through a number of methods. Before families enrolled in the study, a telephone screening form was used to determine the presence of ASD and ADHD in family members. During their infant’s visit to the lab, the parent/caregiver also completed a “Medical and Psychiatric History Interview” (Appendix A) with the researcher. The telephone screening form and this formal interview at a study visit were the primary sources of information about diagnostic status. In addition, we asked for medical updates at each study visit and re-administered the Medical and Psychiatric History Interview at the 2-year timepoint. We also requested diagnostic letters and asked parents to complete the DAWBA (Goodman, Ford, Richards, Gatward & Meltzer, 2000) ASD and ADHD sections and these were reviewed by the senior clinician (TC). In addition, parents completed the Conners (Conners, 2008) (for ADHD) and the Social Communication Questionnaire (Rutter, Bailey & Lord, 2003) and Social Responsiveness Scale (Constantino, 2012; for ASD) on the family member with a diagnosis and where possible all other family members. This information is used to characterise our sample rather than for exclusionary purposes since, in the UK, NHS clinical diagnoses follow a gold-standard procedure including collation of information from parents, teachers and from in-person assessment that is beyond the scope of this study and because it is more accurate than simple questionnaire measures.

## 2. Results

### 2.1 Descriptives

Table S1 Sample sizes of the analyses with EEG data per model. Sleep Onset Latency (SOL); Night awakenings (Awa); Tactile Suppression Index (TSI); Typical likelihood (TL); Elevated Likelihood (EL); EL with a confirmed diagnosis of ASD (EL-ASD+); EL without an ASD diagnosis (EL-ASD-).

| **Model** | Concurrent | | Longitudinal | |
| --- | --- | --- | --- | --- |
| **Outcome** | SOL (10 mo) | Awa (10 mo) | SOL (14 mo) | Awa (14 mo) |
| **Covariates** | Group, TSI (10 mo) | Group, TSI (10 mo) | Group, TSI (10 mo), SOL (10 mo) | Group, TSI (10 mo), Awa (10 mo) |
| **Total n (m:f)** | 58 (31:27) | 58 (31:27) | 50 (27:23) | 49 (26:23) |
| **TL(m:f)/EL(m:f)** | 15(9:6)/43(22:21) | 15(9:6)/43(22:21) | 11(7:4)/39(20:19) | 11(7:4)/38(19:19) |
| **TL/EL-ASD-/EL-ASD+/missing** | 15/34/5/4 | 15/34/5/4 | 11/30/5/4 | 11/29/5/4 |

Table S2 Descriptives of the sleep onset latency (SOL) variable. SE = Standard Error

| **SOL** | **Kolmogorov-Smirnov** | **df** | **Sig** | **Shapiro-Wilk** | **df** | **Sig** | **Skewness (SE)** | **Kurtosis (SE)** |
| --- | --- | --- | --- | --- | --- | --- | --- | --- |
| **5 months** | 0.179 | 66 | 0.000 | 0.840 | 66 | 0.000 | 1.78 (0.26) | 3.64 (0.51) |
| **10 months** | 0.128 | 66 | 0.009 | 0.927 | 66 | 0.001 | 0.78 (0.24) | 0.36 (0.47) |
| **14 months** | 0.218 | 66 | 0.000 | 0.814 | 66 | 0.000 | 1.59 (0.24) | 2.75 (0.47) |

Table S3 Descriptives of infants split by ASD likelihood status and - diagnosis at 36 months. ADOS CSS: Autism Diagnostic Observation Schedule calibrated severity scores; EL: Elevated likelihood; ELC: Early learning component; SOL: Sleep onset latency; TL: Typical likelihood; TSI: Tactile suppression index;

|  | | **ASD-likelihood status** | | **EL group ASD diagnosis** | |
| --- | --- | --- | --- | --- | --- |
|  | | **TL** | **EL** | **EL-ASD-** | **EL-ASD+** |
| **5-month visit** | | | | | |
| n | 25 | | 66 | 48 | 10 |
| Age in Days | 178.42 (13.29) | | 176.08 (19.61) | 175.26 (19.99) | 174.50 (18.47) |
| Sex (M:F) | 18:7 | | 35:31 | 24:24 | 6:4 |
| ADOS CSS | 1.55 (0.69) | | 3.10 (2.25) | 2.83 (1.92) | 3.89 (2.80) |
| Mullen ELC | 85.08 (9.704) | | 83.55 (10.943) | 84.72 (11.78) | 82.60 (15.01) |
| Number of awakenings | 2.44 (1.47) | | 2.00 (1.48) | 1.89 (1.46) | 2.30 (1.95) |
| SOL | 11.15 (10.10) | | 12.42 (10.35) | 10.88 (8.98) | 15.62 (13.43) |
| **10-month visit** | | | | | |
| n | 22 | | 82 | 62 | 10 |
| Age in Days | 318.77 (15.76) | | 318.04 (14.16) | 319.57 (15.05) | 319.40 (9.24) |
| Sex (M:F) | 15:7 | | 46:36 | 34:28 | 6:4 |
| ADOS CSS | 1.53 (0.61) | | 2.97 (2.20) | 2.73 (1.83) | 3.89 (3.14) |
| Mullen ELC | 90.09 (11.05) | | 87.56 (14.66) | 87.61 (14.76) | 87.80 (12.53) |
| Number of awakenings | 1.27 (1.08) | | 1.95 (1.37) | 1.95 (1.40) | 2.00 (1.76) |
| SOL | 7.92 (6.19) | | 11.45 (7.51) | 10.08 (6.42) | 16.25 (10.33) |
| TSI | 0.1412 (0.1407) | | -0.0088 (0.2120) | -0.0098 (0.2246) | -0.0320 (0.1486) |
| **14-month visit** | | | | | |
| n | 19 | | 84 | 65 | 11 |
| Age in Days | 448.16 (17.62) | | 449.56 (18.90) | 451.56 (19.38) | 443.82 (15.68) |
| Sex (M:F) | 12:7 | | 45:39 | 34:31 | 7:4 |
| ADOS CSS | 1.59 (0.71) | | 2.96 (2.16) | 2.69 (1.79) | 4.00 (2.98) |
| Mullen ELC | 80.26 (10.84) | | 76.29 (12.69) | 77.06 (13.81) | 69.27 (7.28) |
| Number of awakenings | 1.00 (1.45) | | 1.87 (1.46) | 1.72 (1.33) | 2.73 (1.74) |
| SOL | 6.46 (7.25) | | 13.18 (11.40) | 12.59 (12.25) | 17.29 (10.15) |

### 2.2 Sleep trajectories based on ASD diagnosis

We reran the GEE analysis for the sleep trajectories by dividing the sample into three groups: Infants at TL, infants at EL that did not receive an ASD diagnosis at 36 months (EL-ASD-) and infants at EL that received a diagnosis of ASD at 36 months (EL-ASD+). First GEE was performed to assess the main effects of groups and visit, before the interaction between groups and visit was added to the model. Then, separate GEEs were run per visit (5-, 10- and 14 months) to assess group differences at each visit. Analyses were done for the number of night awakenings and sleep onset latency separately.

Awakenings

The number of awakenings was significantly affected by visit and decreased with time (Waldχ^2^ = 7.633, p = .022). There were no group effects for TL versus EL-ASD- (Waldχ^2^ = .522, p = .470) or EL-ASD+ (Waldχ^2^ = 1.459, p = .227). However, there was a significant interaction between visit*EL-ASD- (Waldχ^2^ = 10.510, p = .005) and visit*EL-ASD+ (Waldχ^2^ = 9.260, p = .010).

Neither TL versus EL-ASD- (Waldχ^2^ = 2.430, p = .119; EL-ASD-: n = 47) or EL-ASD+ (Waldχ^2^ = 0.044, p = .833, EL-ASD+: n = 10) had a significant effect on the number of awakenings at 5 months. At 10 months El-ASD- had significantly more night awakenings than TL infants (Waldχ^2^ = 4.663, p = .031; EL-ASD-: n = 62), but EL-ASD+ did not (Waldχ^2^ = 2.021, p = .155; EL-ASD+: n = 10). However, at 14 months the results were reversed and EL-ASD+ has significantly more night awakenings than TL infants (Waldχ^2^ = 7.252, p = .007; EL-ASD+ n = 11), while EL-ASD- did not (Waldχ^2^ = 2.563, p = .109; EL-ASD-: n = 64).

Sleep onset latency

In comparison to the TL infants, EL-ASD+ had a significantly longer overall sleep onset latency (Waldχ^2^ = 4.379, p = .036) after controlling for visit, whereas EL-ASD- did not differ significantly from TL (Waldχ^2^ = .227, p = .634). The interactions of both Visit*EL-ASD- and Visit*EL-ASD+ were significant (Waldχ^2^ = 17.173, p < .001; Waldχ^2^ = 9.260, p = .010).

Post hoc tests revealed that TL infants did not differ from EL-ASD- or EL-ASD+ for sleep onset latency at 5 months (EL-ASD-: Waldχ^2^ = .013 , p = .910, p= .812, n= 47; EL-ASD+: Waldχ^2^ = 1.137 , p = .286, n = 10), whereas from 10 months EL-ASD+ had significantly longer sleep onset latency (Waldχ^2^ = 8.185, p = .004; n = 10), which continued at 14 months (Waldχ^2^ = 11.093, p = .001, n = 11). In comparison to TL, EL-ASD- did not have significant different sleep onset latency at 10 months (Waldχ^2^ = 1.791, p = .181, n = 62), but they had a significant longer sleep onset latency at 14 months (Waldχ^2^ = 6.064, p = .014, n = 65).


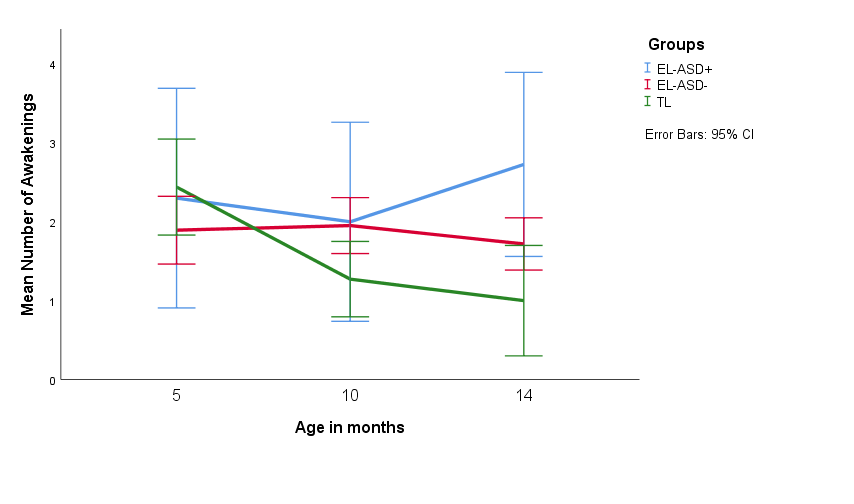


Figure S1 Trajectory of the number of night awakenings. Groups are based on ASD diagnosis at 36 months. TL: infants at Typical Likelihood for ASD; EL-ASD-: Infants at Elevated Likelihood for ASD who were not diagnosed with ASD at 36 months; EL-ASD+: Infants at Elevated Likelihood for ASD who received an ASD diagnosis at 36 months.


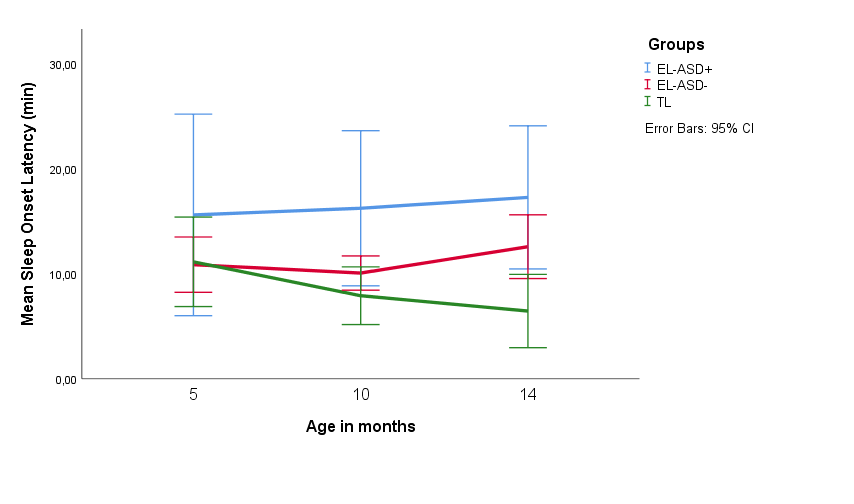


Figure S2 Trajectory of Sleep Onset Latency. Groups are based on ASD diagnosis at 36 months. TL: infants at Typical Likelihood for ASD; EL-ASD-: Infants at Elevated Likelihood for ASD who were not diagnosed with ASD at 36 months; EL-ASD+: Infants at Elevated Likelihood for ASD who received an ASD diagnosis at 36 months.

Table S4 Results of the GEEs of the trajectories of Night Awakenings and Sleep Onset Latency. Infants at elevated likelihood for ASD (EL) were further subdivided into infants with or without a confirmed diagnosis of ASD at 3 years (EL-ASD+ and EL-ASD-, respectively). In the first model, visit (5, 10 and 14 months), group 1 (EL-ASD- vs TL infants) and group 2 (EL-ASD+ vs TL infants) were entered as predictors, then the interactions were added in a second step.

|  | Waldχ^2^ | p-value |
| --- | --- | --- |
| **Number of Awakenings** | | |
| Visit | 7.633 | **.022** |
| EL-ASD- | .522 | .470 |
| EL-ASD+ | 1.459 | .227 |
| Visit*EL-ASD- | 10.510 | **.005** |
| Visit*EL-ASD+ | 9.260 | **.010** |
| **Sleep Onset Latency** | | |
| Visit | 3.199 | 0.202 |
| EL-ASD- | .227 | .634 |
| EL-ASD+ | 4.379 | **.036** |
| Visit*EL-ASD- | 17.173 | **< .001** |
| Visit*EL-ASD+ | 7.438 | **.024** |

Table S5 Results of the GEEs of the trajectories of Night Awakenings and Sleep Onset Latency split by visit (5, 10 and 14 months). Infants at elevated likelihood for ASD (EL) were further subdivided into infants with or without a confirmed diagnosis of ASD at 3 years (EL-ASD+ and EL-ASD-, respectively).

|  | | Waldχ^2^ | p-value |
| --- | --- | --- | --- |
| **Number of Awakenings** | | | |
| 5 months | EL-ASD- | 2.430 | .119 |
|  | EL-ASD+ | 0.044 | .833 |
| 10 months | EL-ASD- | 4.663 | **.031** |
|  | EL-ASD+ | 2.021 | .155 |
| 14 months | EL-ASD- | 2.563 | .109 |
|  | EL-ASD+ | 7.252 | **.007** |
| **Sleep Onset Latency** | | | |
| 5 months | EL-ASD- | .013 | .910 |
|  | EL-ASD+ | 1.137 | .286 |
| 10 months | EL-ASD- | 1.791 | .181 |
|  | EL-ASD+ | 8.185 | **.004** |
| 14 months | EL-ASD- | 6.064 | **.014** |
|  | EL-ASD+ | 11.093 | **.001** |

### 2.3 Associations with tactile repetition suppression without EL-ASD+

We excluded the EL-ASD+ participants (n = 5) and infants who did not come in for the 36-month visit and therefore could not be categorized as EL-ASD+ or EL-ASD-. and reran the GEE with infants at TL and EL-ASD-. Identical to the main analysis, GEE was performed to assess the effects of Tactile repetition suppression, measured at 10 months, on Sleep Onset Latency (SOL) and the number of night awakenings concurrently at 10 months and longitudinally at 14 months in two separate models per sleep measure. Removal of the EL-ASD+ group did not change the results substantively (See Table S5 and S6), suggesting that these infants did not drive the results in the main analysis.

Table S6 Results of the GEEs with sleep measures at 10 months as outcome measures and Tactile suppression index (TSI) and group as predictors. In these models infants at EL-ASD that went on to develop ASD (EL-ASD+ (n = 5)) were excluded resulting in a variable group comprising of infants at TL (n = 15) and infants at EL that did not meet diagnostic criteria for ASD (EL-ASD- (n = 35)).

| **Number of Awakenings** | Waldχ^2^ | p-value |
| --- | --- | --- |
| TSI | .003 | .953 |
| Group | 3.030 | .082 |
| TSI*Group | 1.299 | .254 |
| **Sleep Onset Latency** |  |  |
| Tactile Gating | 6.833 | **.009** |
| Group | 1.378 | .240 |
| Tactile Gating*Group | .275 | .600 |

Table S7 Results of the GEEs with sleep measures at 14 months as outcome measures and Tactile suppression index (TSI), group and sleep measures at 10 months as predictors. In these models infants at EL-ASD that went on to develop ASD (EL-ASD+ (n = 5)) were excluded resulting in a variable group comprising of infants at TL (n = 11) and infants at EL that did not meet diagnostic criteria for ASD (EL-ASD- (n = 30 for number of awakenings; n = 31 for Sleep Onset Latency)).

| **Number of Awakenings at 14 months** | Waldχ^2^ | p-value |
| --- | --- | --- |
| TSI | .012 | .912 |
| Group | 6.014 | **.014** |
| Awakenings at 10 months | 36.805 | **< .001** |
| **Sleep Onset Latency at 14 months** |  |  |
| TSI | .140 | .708 |
| Group | 11.877 | **.001** |
| SOL at 10 months | 17.884 | **< .001** |
